# Inflammatory Myo-Fibroblastic Tumor of the Gallbladder with Multivisceral Involvement: Successful Treatment with Radical Surgery

**DOI:** 10.1155/2023/1909570

**Published:** 2023-06-21

**Authors:** Kwanchanok Losuwarat, Vor Luvira, Vasin Thanasukarn, Theerawee Tipwaratorn, Piti Ungarreevittaya

**Affiliations:** ^1^Department of Surgery, Faculty of Medicine, Khon Kaen University, Khon Kaen, Thailand; ^2^Department of Pathology, Faculty of Medicine, Khon Kaen University, Khon Kaen, Thailand

## Abstract

Inflammatory myo-fibroblastic tumor (IMT) of the gallbladder is an extremely rare condition. Only seven cases have been reported. All of these were presented either with polyp/mass inside the gallbladder or gallbladder wall thickening, involving just one adjacent organ. We herein present a case of IMT of gallbladder presenting with a huge mass replacing the gallbladder with multiple organ involvement, successfully treated by *en bloc* multivisceral resection. Moreover, we have compared it with the characteristics of all reported cases of IMT of the gallbladder.

## 1. Introduction

Inflammatory myo-fibroblastic tumor (IMT) is a rare variant of a rare condition, inflammatory pseudotumor. Although IMT has been described since 1939, the nature of this disease remains unclear, with unknown true etiology, unknown natural history and unknown appropriate definite treatment. It is now designated as an intermediate-grade malignancy, according to WHO classification [1], because of its potential to recur and metastasize, combined with evidence of chromosomal rearrangement in the tumor. Diagnosis of this condition is mainly based on pathological characteristics of fibroblast or myofibroblast proliferation with inflammatory cell infiltration [2]. Even though this tumor can be found throughout the body and in the gastrointestinal tract, IMT of the gallbladder is extremely rare. There have been seven reported cases of IMT of the gallbladder. All of these were presented with polyp/mass inside the gallbladder or gallbladder wall thickening, involving just one adjacent organ. Herein, we would like to present a case of IMT of the gallbladder presenting with a huge mass replacing the gallbladder with multiple organ involvement, successfully treated by en-bloc multi-visceral resection.

## 2. Case Presentation

**Figure 1:**
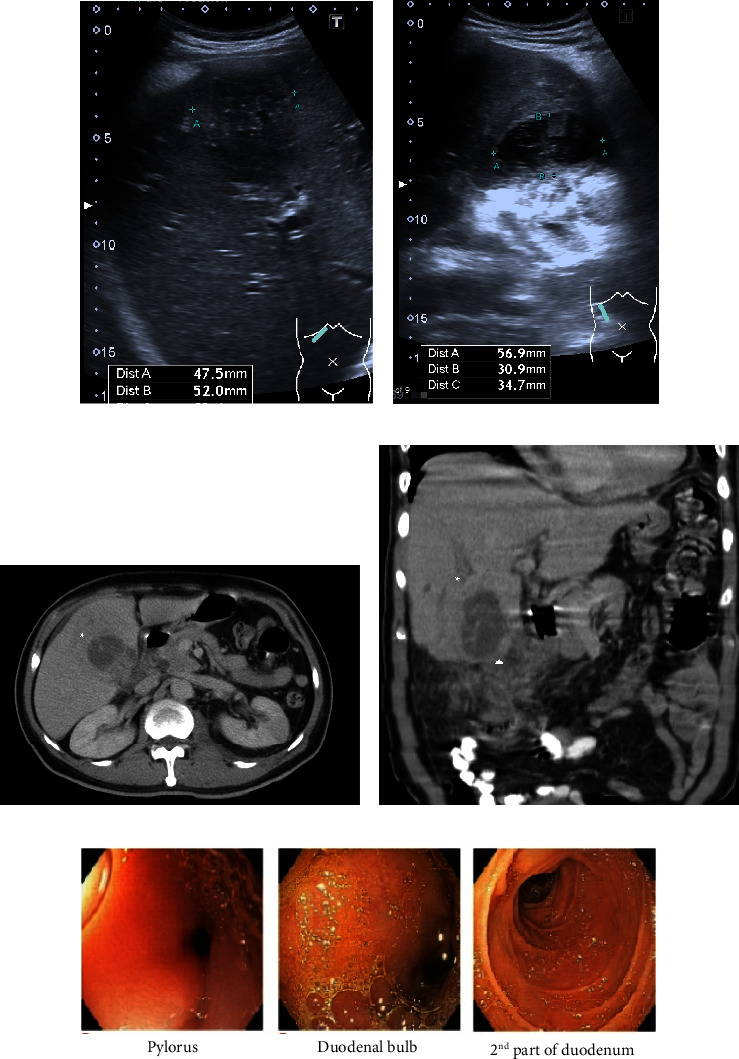
Preoperative investigations. (a, b) Ultrasonography of abdomen showed a heteroechoic gallbladder mass with suspected liver invasion. (c, d) CT whole abdomen revealed a hypodensity mass replacing the gallbladder, invading segment 4, 5 of the liver (asterisk), right-sided colon (triangle) and suspected duodenal invasion. (e) EGD findings showed no mucosal-invading lesions in stomach and duodenum.

A 63-year-old man presented with significant weight loss, from 68 kg to 60 kg (11.2% in 1 month), and abdominal discomfort for 1 month. Physical examinations were unremarkable. His liver function tests were within the normal limit, except for low albumin/globulin ratio of 3.2/5.1. Ultrasonography of the abdomen showed a hetero-echoic gallbladder mass invading the liver (Figures 1(a) and 1(b)). He subsequently underwent computed tomography of the abdomen, which revealed a huge hypodensity mass replacing the gallbladder, invading the hepatic flexure of the colon and with suspected duodenal invasion. There was no significant lymph node enlargement or distant metastasis (Figures 1(c) and 1(d)). For evaluation of gastrointestinal tract involvement, upper and lower gastrointestinal tract endoscopies were carried out, revealing no mucosa invasion (Figure 1(e)).

We had made the discussion, in our team, regarding the possibility of benign conditions, such as xanthrogranulomatous cholecystitis, role of preoperative tissue biopsy and neo-adjuvant treatment. Presence of benign condition on biopsy results, was unable to ensure that this whole mass can be assumed as benign condition and be treated successfully by medication only. On the other hand, if the mass was malignancy, percutaneous biopsy might increase risk of tumor seeding, and, in addition, neo-adjuvant did not ensure tumor response. The tumor might become unresectable after systemic treatment administration. Since there was no evidence of lymph node involvement or distant metastasis, despite local advancement of the mass, we considered it as some kind of gallbladder tumor with good biology, which was resectable both technically and oncologically, and, consequently, may gain benefit from surgical resection. Taken together, the operation was carried out; we firstly performed laparoscopic diagnosis to eliminate the chance of preoperatively undetectable small peritoneal metastases. Then we proceeded with laparotomy, and we found a gallbladder mass invading the liver, the hepatic flexor colon, the lateral wall of the 2^nd^ part of the duodenum, and, unexpectedly, the common hepatic duct and the portal pedicle of the right lobe liver. We, therefore, performed *en bloc* extended right hepatectomy, extended right hemicolectomy, with resection of the gallbladder, extrahepatic bile duct and a portion of the 2^nd^ part of the duodenum. We started the operation from the colon toward the liver because of technical simplicity. At our center, we prefer to perform ileal-colon anastomosis rather than colon-colon anastomosis, therefore we opted to perform right hemicolectomy rather than segmental colon resection. After the colon had been transected, the 2^nd^ part of the duodenum was fully visualized, and we found that just only lateral wall of the 2^nd^ part of duodenum got involve by the tumor. Instead of pancreaticoduodenectomy, therefore, we performed resection of duodenal wall by sharp opening into the duodenal lumen using the Metzenbaum scissors, to ensure the adequacy of surgical margin, then we repaired the duodenal defect using an absorbable, synthetic braided suture, in continuous locking fashion. We had an opportunity to carefully re-assessed the relation between the tumor extension and hepatoduodenal ligament, and found that it was impossible to performed the safety dissection of the adherence between the tumor and right-side of the hepatoduodenal ligament. We, therefore, performed extended right hepatectomy with bile duct resection. Biliary reconstruction was performed using absorbable, synthetic braided suture, in interrupted fashion.

**Figure 2:**
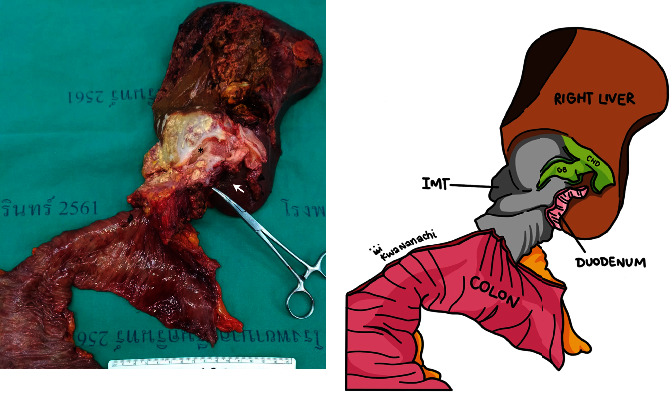
Intraoperative findings. (a) Surgical specimen (b) diagram demonstrating parts of the specimen. The specimen consisted of the gallbladder, the right lobe of the liver, extrahepatic bile duct, part of the 2^nd^ part of the duodenum (arrow), and right-sided colon. An ill-defined inhomogeneous firm mass with whitish and yellowish fleshy cut surface originated from the contracted gallbladder (asterisk), invading segment 4, 5 of the liver, 2^nd^ part of the duodenum, proximal part of the transverse colon, and the common hepatic duct.

**Figure 3:**
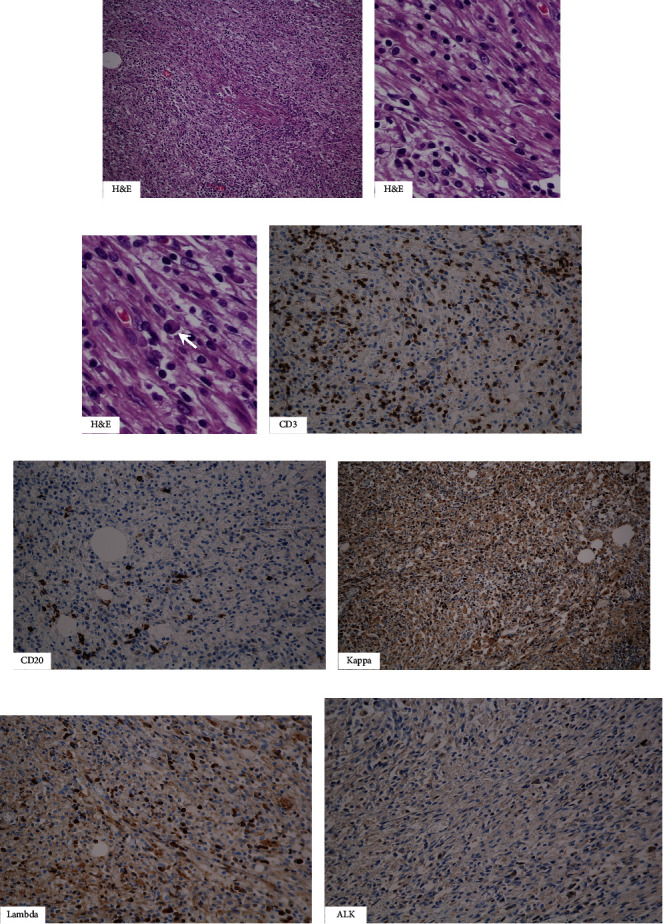
Micrographs. (a–c) H&E stain showed fibroblast-liked spindle cells, small lymphocytes, and plasma cells (arrow) in the adventitia of the gallbladder. (d, e) CD3 and CD20 were scatter stained, indicating a reactive condition. (f, g) Kappa and Lambda were diffusely stained indicating no hematologic malignancy. (h) ALK stain was negative.

A surgical specimen showed an ill-defined inhomogeneous firm mass with whitish and yellowish cut surfaces occupying over the fundus and body of the gallbladder (Figure 2). Histopathology revealed fibroblast-like spindle cells, small lymphocytes and plasma cells (arrow) in the adventitia of the gallbladder (Figure 3(a)–3(c)), whereas mucosa and muscularis propria appeared normal. On immunohistochemical studies, CD3, CD20, Kappa and Lambda were scatter stained (Figures 3(d)–3(g)). Anaplastic lymphoma kinase (ALK) stain was negative (Figure 3(h)). The pathological diagnosis was IMT of the gallbladder invading the right lobe of the liver, the hepatic flexor colon, the 2^nd^ part of the duodenum and the common hepatic duct. The postoperative course was uneventful. He is on regular follow up and has been disease free for 2 years.

## 3. Discussion

We have described a case of IMT of the gallbladder with multiple organ involvement, successfully treated by *en bloc* multi-visceral resection. The patient still has disease-free status. We have searched extensively through medical databases, using the keywords; IMT OR “inflammatory myofibroblastic tumor” OR “pseudotumor” OR “inflammatory fibroblastic” AND gallbladder, and found seven reported cases of IMT of gallbladder. In all of these, the disease mainly involved the gallbladder and one adjacent organ [3–9] (Table 1).

All reported cases of IMT of the gallbladder, including our case, share many similar unique features. Although IMT is usually found in children and young adults, the IMT of the gallbladder mostly presented in late middle age. The youngest reported case of IMT of gallbladder was in a 35 year-old [4]. All cases were symptomatic, with abdominal pain being the most frequently found symptom. The etiology of this condition remains unknown; none of the reported cases identify the exact cause of the patient's condition. Our patient experienced some constitutional symptoms, fever and weight loss; those might either indicate the preexisting inflammatory condition or originate from the tumor. The indolent, non-aggressive nature of this condition was supported by the reported cases. None of these had metastatic disease, and only one case experienced a recurrence [8]. In our case there were no metastases, even though the tumor was locally advanced. This nature allows the surgeon to perform a radical surgery for this condition. Although non-surgical intervention has been reported for treatment of IMT in many organs, we believe that surgical resection is the optimal treatment for IMT of the gallbladder because (i) It is unable to exclude malignant gallbladder tumor for preoperative investigation, (ii) All reported cases experience some symptoms, (iii) There is not an effective non-operative treatment, and (iv) Its non-metastatic, slow-progression nature makes resection the optimal treatment. Nowadays, postoperative definite diagnosis of IMT of the gallbladder is based solely on the histopathological characteristics on pathological examination. Since the anaplastic lymphoma kinase (ALK) gene is believed to account for the development of IMT, positive immunohistochemistry (IHC) staining for ALK is considered to be one of diagnostic methods for IMT [10]. Among five reported cases where IHC for ALK was performed, only one case showed positive staining [9], whereas the remaining showed equivocal and negative results. However, due to similarities in patients, tumor and histological characteristics, we believe that all reported cases might be the same entity, and IHC for ALK may lack the sensitivity to detect IMT of the gallbladder. Patients with ALK-negative IMT tumor should be further examined for gene arrangement by fluorescence *in situ* hybridization, or ROS-1 gene fusion.

To the best of our knowledge, our case was the first reported case of IMT of gallbladder which presented with a mass replacing the gallbladder, and involved more than one adjacent organ. It implies that such an inflammatory tumor can present as a large, locally invasive lesion. We still suggest that gallbladder cancer should be considered first when a physician encounters either a large gallbladder mass or a mass replacing gallbladder lesion. Regarding the treatment of this patient, the decision to perform curative intent surgery was quite difficult. To archive R0 resection in this case, extensive surgical resections were required, including extensive liver resection, resection of part of the duodenum, and hemicolectomy, which carried a high risk of morbidity. Given the patient's preferences, good performance status, symptoms of the tumor, and evidence of good biology of the tumor (no sign of lymphatic or distant spreading), we therefore performed such an aggressive operation. Although the patient still has a good outcome at 2 years post-operation, we believe that all patients with IMT require a longer follow-up period to ensure curation.

## 4. Conclusion

All reported cases of IMT of the gallbladder share many unique features, which differ from IMT of other organs. All cases were in the middle-aged, were symptomatic, and involved no more than one adjacent organ. We have reported the first case of IMT of gallbladder with multiple organ involvement, successfully treated with multi-visceral resection.

## Figures and Tables

**Table 1:** Reported cases of inflammatory myo-fibroblastic tumor of gallbladder.

Author	Year	Gender	Age	Preoperative diagnosis imaging finding	Operation	Organ involvement	Size (cm)	ALK	Long term outcome
Behranwala [3]	2005	Female	51	Acute cholecystitis (*Wall thickening*)	Cholecystectomy transverse colectomy	Gallbladder transverse colon	12	Equivocal (IHC)	Disease-free for 6 months (at least)
Muduly [4]	2012	Female	35	Gallbladder cancer (*Mass inside gallbladder*)	Radical cholecystectomy liver s4, 5 resection lymph node dissection	Gallbladder	NA	NA	Disease-free for 2 years (at least)
Özsan [5]	2013	Male	66	Gallbladder cancer (*Mass inside gallbladder*)	Radical cholecystectomy liver s4, 5 resection	Gallbladder	NA	NA	NA
Badea [6]	2014	Female	65	(i) Gangrenous cholecystitis (ii) Gallbladder cancer (*Wall thickening*)	Radical cholecystectomy liver s4, 5 resection lymph node dissection	Gallbladder liver	6.5 × 5.2 × 4	Equivocal (IHC)	Disease-free for 3 months (at least)
Sinha [7]	2017	Female	36	NA (*Wall thickening*)	Radical cholecystectomy resection of 1st part of duodenum and pylorus lymph node dissection	Gallbladder duodenum: 1^st^ part pylorus	NA	NA	NA
Maruyama [8]	2017	Female	63	Gallbladder tumor (*Gallbladder mass*)	Cholecystectomy	Gallbladder	NA	Negative (IHC and FISH)	Recurrence at 13 months
Yamada [9]	2018	Male	50	(i) Atypical gallbladder cancer (ii) Non-neoplastic polyp (*Polypoid mass*)	Cholecystectomy liver resection	Gallbladder	2	Positive (IHC)	Disease-free for 6 years (at least)
Losuwarat (present study)	2022	Male	63	Gallbladder tumor (*Mass replacing gallbladder*)	Cholecystectomy extended right hepatectomy bile duct resection right hemicolectomy resection of 2^nd^ part of duodenum lymph node dissection	Gallbladder liver common bile duct right hepatic duct duodenum 2^nd^ part ascending colon	9 × 7	Negative (IHC)	Disease-free for 2 years (at least)

## Data Availability

The patient data used to support the findings of this study are included within the article.
